# Development and Validation of the Celiac Disease-Children’s Activities Report (CD-Chart) for Promoting Self-Management among Children and Adolescents

**DOI:** 10.3390/nu9101130

**Published:** 2017-10-17

**Authors:** Sonya Meyer, Sara Rosenblum

**Affiliations:** The Laboratory of Complex Human Activity and Participation, Department of Occupational Therapy, Faculty of Social Welfare & Health Sciences, University of Haifa, Haifa 3498838, Israel; rosens@research.haifa.ac.il

**Keywords:** celiac disease, food-related activities, ICF-CY, questionnaire

## Abstract

Adherence to a restrictive gluten-free diet is the only known treatment for celiac disease (CD). Children and adolescents with CD encounter challenges while managing the diet in daily activities. Understanding their participation characteristics is lacking. The aim was to describe the development and validation process of the Celiac Disease-Children’s Activities Report (CD-Chart). The final CD-Chart includes nine food-related activities that are measured by six core dimensions: frequency, preference, preparation, involvement, help, and self-determination. Participants were 126 children (8–11 years) and adolescents (12–18 years) with CD, and 30 healthy matched controls. Factor analysis was performed and psychometric properties were measured. Content and face validity was established and the CD-Chart showed adequate internal consistency as measured by the preference dimension (α = 0.81). Factor analysis revealed two distinct factors, social environment and close family environment. Construct validity demonstrated that the group with CD required significantly more pre-preparation for food-related activities than controls, (t(38) = 76.25, *p* < 0.001) and further differences as well as similarities were found between groups. Primary results indicate that the CD-Chart may serve as a practical tool for acquiring information about participation characteristics in food-related activities, strengths and challenges of children and adolescents with CD, to promote self-management, diet adherence, and well-being.

## 1. Introduction

Celiac disease (CD) is a chronic small-intestinal, immune-mediated enteropathy precipitated by exposure to gluten (wheat, rye and barley) that is estimated to affect approximately 0.3–1.3% of the population [1,2]. Lifelong adherence to a strict gluten-free diet (GFD) remains the only known effective treatment for CD and adherence specifically among children and adolescents present daily challenges [3,4,5]. 

Assessing the level of adherence does not fully express the negative impact of managing a GFD on quality of life (QOL), because it does not reveal the actual daily challenges entailed in adhering to the diet [6]. The results of various disease-specific questionnaires or items added to nonspecific measures of QOL or health-related QOL of children and adolescents with CD have been previously described (e.g., [7,8,9,10]). The areas explored in such studies mainly consider feelings concerning CD, emotional difficulties, anger, stigma, self-perception, and concerns in the home, and the school and social environments in food-related situations (e.g., [9,11,12,13]). However, despite the beneficial outcomes of these measures, they do not explicitly consider the central concepts offered by the World Health Organization (WHO) [14] for capturing implications of a disease on the individual’s daily function, the International Classification of Functioning, Disability and Health (ICF) [14], and the children and youth version (ICF-CY) [15]. This classification describes health conditions through the lens of functioning, while assessing interactions between body functions and structures, activities and participation; it also considers environmental and personal factors [15]. The ICF and ICF-CY have been adapted for varied health conditions and measurements developed and utilized to complement the medical diagnoses, including various chronic disorders [16], yet not for CD. 

Participation is defined as a person’s involvement in life situations and its inclusion in the ICF represents a shift from the biomedical model towards a social model of disability in which the environment plays an important role [17]. Participation in meaningful occupations has a direct impact on health and QOL [18]. Previous research has found correlations between health-related QOL and social aspects of participation in specific food-related activities among children with CD [19]. Further examination of literature in light of the ICF-CY [15] revealed gaps in the understanding of activities and participation characteristics of daily life with CD and the need to consider their self-management abilities as a contributing factor to their QOL was highlighted [20,21,22]. Self-management involves active patient participation in caring for, and living well, with a chronic health condition [23,24]. Self-management is comprised of five core cognitive skills: problem solving, decision-making, resource utilization, forming of patient/health provider partnership and taking action [23]. These cognitive skills, are included in the ‘body function’ component of the ICF-CY classification [15]. 

Various studies have described participation in the daily activities of children and adolescents (e.g., [25,26,27]). However, an assessment process that is oriented towards promoting self-management abilities and addresses how this population actually participates in daily food-related activities is lacking [15,19,28,29]. This gap led to the development of the Celiac Disease-Children’s Activities Report (CD-Chart), founded on the analysis of the needs of children, adolescents with CD, and their parents via focus groups [30]. The objective of the CD-Chart is to capture the unique participation and self-management characteristics of children and adolescents with CD [20] in order to provide an accurate understanding of where their problems lie and to map out of their performance to possibly serve as a basis for planning interventions [31]. 

The purpose of this article is to describe the three-phased development process of the CD-Chart and to examine its internal reliability and validity among children and adolescents with CD, aged 8–18 years, in order to incorporate this tool into future interventions for promoting self-management.

## 2. Phase I: CD-Chart Development and Item Selection

### 2.1. Methods

The questionnaire was originally developed in Hebrew and has undergone a process of valid translation to English and back-translation. The construction of the CD-Chart was founded on four main bodies of knowledge and oriented towards self-management needs: (1) analysis of previously-conducted focus groups [30]; (2) adaptation of structure and dimensions of the Children’s Leisure Assessment Scale (CLASS) [27]; (3) the ICF-CY concepts [15]; and (4) Occupational Therapy Practice Framework: Domain and Process [32]. Expert review of the selected items was conducted by a panel of thirteen professional and lay experts to establish the questionnaire’s content validity. Content validity refers to the extent to which the items on a measure sufficiently represent the number and scope of the relevant issues and the clarity of items [33].

### 2.2. Phase I Results

Based on previously performed focus group outcomes [30], eighteen food-related activities that occur in daily life were chosen. However, following a pilot administration of the questionnaire, the items were reduced to nine activities that best reflected experiences of children and adolescents aged 8–18 in everyday life, in order to adapt to participants attention span. Reduction was achieved by integrating items together and by incorporating examples in place of separate items. The nine items are: (1) participating in preparing meals at home; (2) participation in extended family meals/events; (3) participating in meals during family vacations (local and abroad); (4) participating in eating out with friends; (5) participating in food served at a friend; (6) participating in meals at an overnight camp/trip; (7) participating in eating treats handed out by the teacher; (8) participating in special food activities at school; and (9) participating in meals during overnight school field trips. 

The CD-Chart is standardized in its construct and specificity of administration and scoring method. As the CD-Chart is patient-tailored [34,35], the scores do not generate a total score and is not a norm-referenced measure. Rather, the CD-Chart assists in identifying strengths and weaknesses as a basis for establishing client-centered goals to promote self-management [36]. The CD-Chart includes the following quantitative dimensions in order to assess each of the nine food-related activities. 

*Activity* defines whether the child or adolescent engages in the activity and can be scored as follows: 1 = participates or 0 = does not participate. 

*Frequency* defines how often the child or adolescent participates in the activity and was measured on a 4-point Likert scale; 1 = once a year/every few months, 2 = ONCE or twice a month, 3 = once or twice a week, and 4 = every day.

*Preference* measures how much the child or adolescent likes participating in the activity as rated on a 10-point scale ranging from 1 (not like at all) to 10 (like very much). 

*Preparation* defines whether participation in the activity requires any special advance preparation and is scored as follows: 0 = requires no preparation or 1 = requires preparation. 

In the case of an answer that preparation is required, the following questions are asked: 

*Who?* A follow-up question includes a description of who helps with the preparation (e.g., help myself, help from parents, other family members, friends, guide, coach, school, teacher). 

*Involvement* states the level of the child’s or adolescent’s involvement in the preparation process rated on a 5-point Likert scale; 5 = independent, 4 = involved in cooperation with someone else, 3 = receive some help, 2 = I am informed but do not perform independently, and 1 = dependent.

*What?* A follow-up question that describes what type of help is received (e.g., receive instructions on how and what to do, have someone read out the ingredients, have someone ask questions, have someone check on the Internet, have someone prepare food, prepare food together with someone). 

*Self-determination* expresses how important it is to the child to perform independently and is scored on a three-point Likert scale; 3 = It’s important to me to perform independently, 2 = I don’t mind getting help, and 1 = I prefer others do it for me.

Additional qualitative supplementary structured questions referring to supports, barriers and self-strategies were presented to the participant for further clinical and research use. Any additional remarks or experiences were also recorded.

In order to establish the CD-Chart’s expert validity, a panel of experts was recruited. The panel included specialists in the field of children and youth with expertise or knowledge about CD and was comprised of four occupational therapists, one educational psychologist, one social worker, and one registered dietician. Additionally, one adolescent and two mothers who participated in the previously performed focus groups reviewed the CD-Chart. Experts confirmed the relevance of the items. Following, the experts’ feedback, comments, mutual consultation and agreement, examples were added to two of the activities and minor modifications were made in wording in order to assure clarity, thus determining the final version of the CD-Chart. 

## 3. Phase II—Analysis of Reliability and Validation

### 3.1. Methods

In order to establish the CD-Chart’s reliability and validity, the final version of quantitative and qualitative questions was administered by the first author during an interview session at the family’s home. Parents completed an online questionnaire that included demographic and health information including questions about functional developmental background, psychological, and/or allied health professional assessment or therapy. 

#### 3.1.1. Participants

The study sample were 126 children and adolescents diagnosed with CD. The sample size was calculated with the G*Power statistical program, based on a moderate effect size (α < 0.5; power = 0.80) [37]. The University of Haifa, Faculty of Social Welfare & Health Sciences Ethics Committee on Research with Human Participants approved administration of the CD-Chart as part of a larger study and all participants were provided informed consent (Approval number 026/15, 18 January 2015). Participants were recruited via the Israel Celiac Association, local online support groups and social media. The inclusion criteria for the study group were children and adolescents aged 8–18 years with confirmed diagnosis of CD by a physician for no less than six months prior to the study and proficiency in the Hebrew language. Children diagnosed with physical and/or neurological disabilities were excluded. 

Participants included 82 girls and 44 boys, aged 8 to 18 years (*M* = 12.33 years, SD = 2.85). All participants were diagnosed with CD by biopsy (89.7%) or blood tests (10.3%). Most were diagnosed before the age of 11 years (88.9%) and 67.5% were diagnosed over three years ago. According to the information reported by the parents about the children’s development and functioning, almost all the participants (97.6%) always adhere to a GFD and only three often adhere to the diet. None of the participants were reported to have common co-morbidities such as anxiety or eating disorders. 

All the participants were Jewish and studied in mainstream schools in Grade 2 to Grade 12 (*M* = 6.56, SD = 2.95), 54.0% in elementary school, 24.6% in middle school and 21.4% in high school. The majority lived in the city (69%) and the remaining lived in various community settings. The family’s income level was mostly high (63.5%) or average (25.7%), and the mothers (*M* = 45 years, SD = 4.79) were all school graduates (*M* = 16.33 years, SD = 2.75). 

#### 3.1.2. Data Analysis

Data were analyzed with SPSS version 23 (IBM Corp, Armonk, NY, USA). Descriptive statistics were used to describe the CD group. Cronbach’s alpha was calculated to establish reliability and a level of α > 0.65 was defined as acceptable [38]. Exploratory factor analysis with varimax rotation was applied to the item responses to assist in the development, refinement, and evaluation of the CD-Chart [39]. Pearson chi-square values were calculated to determine correlations. Multivariate analysis of variance (MANOVA) was used to test for group differences and univariate analyses of variance (ANOVAs) to examine the source of the significance. Pearson’s chi-squared test was calculated to compare who provides the children and adolescents with help and what type of help they receive.

### 3.2. Phase II Results—Construct Validity

#### 3.2.1. Internal Consistency

The CD-Chart’s internal consistency was determined by calculating Cronbach’s coefficient α (α = 0.81) for all nine items across the preference of participating in the activities domain by the 126 participants with CD. 

#### 3.2.2. Exploratory Factor Analysis

Factor analysis performed on the preference domain data revealed two distinct factors was made up of seven items (Table 1). The first factor (five items) combined together activities entailing participation in food-related activities in the social environment. The second factor (two items) included participation in food-related activities with close family members. Item 6 (participating in meals at overnight camp/trip) and item 9 (participating in meals during overnight school field trips) were excluded from the factor analysis as they refer to activities that are age irrelevant to most of the younger participants and, therefore, the number of participants was insufficient to perform this analysis. 

Adequate reliability of the CD-Chart as measured on the preference dimension of Factor 1 was achieved (α = 0.66) and correlation measures in Factor 2 were found to be weak, yet significant, *r* = .20, *p* < 0.05, therefore justifying their correlation. Although items 6 (participating in meals at overnight camp/trip) and 9 (participating in meals during overnight school field trips) were not included in the factor analysis, correlations were calculated because, while the younger group rarely goes on overnight school trips, some do go on trips with their youth movement. The results showed a good theoretical relationship between these items, *r* = 0.49, *p* < 0.001 thus defining “trip environment” as a third component.

## 4. Phase III—Further Validation Analysis

### 4.1. Comparing the CD-Chart of Children with and without CD

Based on the factor analysis results, the CD-Chart’s ability to distinguish between 60 participants with and without CD was examined.

#### 4.1.1. Method

Thirty participants from the entire sample with CD were matched by age, gender, and social environment to a control group of 30 healthy participants without CD or other chronic health conditions. The participants with CD were conveniently chosen among those living in the central region of the country and included two or three participants of each age between eight and 18 years. The control group consisted of friends or neighbors of the participants with CD. Participants in both groups were Jewish and studied in mainstream schools and both groups had equal representation of girls and boys (50%). No significant differences were found between the groups in participants’ age (*M* = 13.01, SD = 3.07), or grade (*M* = 7.12, SD = 3.04); in the family’s income level that was mostly high (78.3%) or average (21.7%), the mother’s age (*M* = 47 years, SD = 3.96) or the mother’s education level (*M* = 15.77 years, SD = 2.10). The control group underwent the same the process of the CD-Chart administration by the first author at the family’s home and parents completed an online questionnaire that included demographic and health information. 

#### 4.1.2. Results

The first four dimensions’ mean scores were calculated across all nine items and compared between the two groups. The preparation dimension showed that the entire CD group required preparation and the control group require no preparation. Thus, the following dimensions (involvement and self-determination) were excluded from further analysis when comparing these two groups. 

MANOVA results yielded significant differences between the groups (*F*(3,56) = 7.05, *p* < 0.001, η *p*^2^ = 0.27). As presented in Table 2, the following ANOVAs found significant differences in two of the three dimensions. The CD group participated in significantly less activities and liked the participation less. Nonetheless, no significant difference was found between the groups in the frequency in which they participated in the activities. Further comparison was calculated for each of the two factors (social environment and close family environment) and the additional component (trip environment). 

##### Social Environment 

MANOVA results indicated a statistically significant difference in participation characteristics between the two groups (*F*(3,56) = 7.43, *p* < 0.001, η^2^ = 0.28). As presented in Table 3, ANOVA analysis indicated that children with CD participate in significantly less activities and like participating significantly less than the controls. 

##### Close Family Environment 

Results of the MANOVA indicated significant differences between the groups (*F*(3,56) = 1.11, *p* = 0.355, η^2^ = 0.06). The following ANOVAs revealed that the source of the significance is in the CD group’s need for advance preparation (Table 3). 

##### Trip Environment

MANOVA results indicated significant differences between the groups (*F*(3,43) = 3.36, *p* = 0.027, η^2^ = 0.19). The following ANOVAs showed that the CD group liked participation in these activities significantly less than the control group. 

### 4.2. Comparing the CD-Chart of Children with CD (8–11 Years) and Adolescents with CD (12–18 Years)

#### 4.2.1. Method

To further establish validity, the entire group of participants with CD was divided into two sub-groups: (1) children aged 8–11 years (*n* = 61); and (2) adolescents aged 12–18 years (*n* = 65), based on the acceptable definition of adolescence in Israel [40].

#### 4.2.2. Results

Mean scores of the six dimensions were calculated across all nine items and the results of the MANOVA indicated significant differences between the groups (*F*(6,119) = 14.25, *p* < 0.001, η^2^ = 0.42). The following ANOVAs found significant differences in five of the six dimensions (Table 4). The children participated in significantly less activities and less frequently than the adolescents and the adolescents liked participating significantly less than the children.

Further comparison was calculated for each of the two factors and the additional component and significant differences are presented in Table 5. 

##### Social Environment 

Results of the MANOVA indicated a significant difference in participation characteristics between the groups (*F*(6,119) = 5.73, *p* < 0.001, η^2^ = 0.22). Examination of each dimension within this factor indicated that the children with CD liked participation in these activities significantly more than the adolescents with CD. However, the adolescents were significantly more involved in the preparation process and showed significantly higher self-determination than the children (Table 5). 

##### Close Family Environment 

MANOVA results indicated significant differences between the groups (*F*(6,118) = 6.31, *p* < 0.001, η^2^ = 0.24). As indicated in Table 5, while children with CD participated significantly less often in the activities than adolescents, the adolescents with CD were significantly more involved in the preparation and showed significantly higher self-determination (Table 5) in comparison to the children. 

##### Trip Environment 

The MANOVA results indicated significant differences between the groups (*F*(5,87) = 13.64, *p* < 0.001, η^2^ = 0.44). The children participated in significantly fewer activities and less frequently than the adolescents (see Table 5). The adolescents had significantly lower preference for these activities than the children yet were significantly more involved in the preparation process.

## 5. Discussion

The CD-Chart was developed in response to a recognized need to provide a valid and reliable tool for the evaluation of unique participation characteristics among children and adolescents with celiac disease who are required to adhere to a life-long GFD [19]. Indeed, the findings show that the CD-Chart items have good internal reliability for measuring the chosen food-related activities (α = 0.81). 

The two factors found for participation preference in the current study were the social environment and the close family environment. These factors correspond with the meaning of participation as a direct result of the interaction between personal and environmental factors [32,41]. 

More specifically, previous literature has emphasized the important role of specific environments such as at home, out of the home, school, and with friends and diverse experiences in different environments among children and adolescents with CD has been described [8,13,42,43,44,45]. However, the CD-Chart can differentiate not only between activities in relation to environments, but the activities were more accurately characterized by the relationship with the people involved in the participation. Indeed, according to the ICF-CY, interpersonal interactions and relationships with people appear as one of the components that needs to be taken into account with reference to activities and participation. Among the many ICF-CY sub-classifications that are included in the interpersonal interactions and relationships chapter are relationships with children, with parents, and with siblings. These kinds of relationships are in line with the close family environment factor found in this study. Whereas other ICF-CY sub-classifications, for instance the extended family, friends and formal relationships are pursuant to the CD-Chart social environment factor [15]. Thus, these factors accentuate the significant roles of different relationships according to the ICF-CY activities and participation, when examining characteristics of developing children and the influence of the environment [15,17]. 

Given that the ICF-CY also defines the health condition, the CD-Chart indeed differentiated between participants with and without CD. It was interesting to find similar frequencies of participation alongside different preference ratings. Recognition of these subtle differences that are unique to the children and adolescents with CD was accomplished by incorporation of the ICF-CY concepts that can capture the unique nuances of functioning. Such subtle differences in participation are reflected by the lower scores that were evident only in the social environment factor and the trip environment component, yet not in the close family environment. The age range in this study encompassed the developmental stage of childhood years approaching adolescence, during which the value of peers becomes increasingly important and the desire to belong increases [46]. As adolescence progresses, they strive to fit in and their peer relationships offer them social integration, a sense of belonging and acceptance [47]. Thus, the similar CD-Chart scores in frequency of participation alongside the activity scores, may possibly reflect the limitations that the group with CD experience and therefore their choice to avoid participation in some food-related activities. Additionally, the lower preference scores, albeit similar participation frequency characteristics, may possibly reflect the many burdens experienced by that those diagnosed with CD, when eating out, travelling, socializing with friends, and at school [48]. These described interactions that are revealed by the CD-Chart that reflect specific food-related activities, may be conveyed in the required self-management characteristics and thus impact participation in daily activities [23].

As children grow from childhood into early adolescence, their participation in daily life activities takes place in wider, more diverse, circles and away from home [49]. The sensitivity of the CD-Chart to developmental factors was highlighted in the varied features that were found in the differences between the younger and older groups with CD. The developmental process of the child’s transition from dependency in infancy towards physical, social and psychological maturity and independence in adolescence is dynamic [15]. Generally, young children require more mediation and supervision while participating in basic daily activities. However, as they develop, they need sophistication and refinement of daily activities, such as managing and preserving a healthy lifestyle [32,50]. During the critical transition to adolescence, several main developmental experiences occur such as: striving for independence, gaining of control, development of identity, acquisition of skills needed to carry out adult relationships and roles, and the capacity for abstract reasoning [51,52]. The distinction in preference of participation that was recognized in the CD-Chart as the children grow into adolescence might reflect the complexity of this transitional stage. Furthermore, this difference may be due to their increased responsibility and commitment to sticking to a GFD during this developmental stage, alongside the shared responsibility with the parents [53]. The ability of the CD-Chart to identify the subtle changes that occur as children transition from childhood to adolescence is important in order to appropriately facilitate the encouragement of responsibility for self-management of the health condition [53]. 

There are some limitations to this study. Confirmation of CD diagnoses was based on parental report of biopsy or blood tests and adherence to a GFD was obtained via parental and self-report. Future studies should consider collaboration with gastroenterologists and dieticians who specialize in CD in order to obtain and consider additional information, such as different clinical representations of CD. Due to technical limitations of administrating the CD across the country, the control group was limited to only 30 participants. Nevertheless, the control group was matched and covered the full age range (8–18) and this amount was sufficient for statistical validation purposes. In addition, participants were recruited via the local celiac association and social media and, therefore, may not represent the entire local celiac population or those in other countries. 

## 6. Conclusions

The primary results indicate that the CD-Chart may serve as a practical tool for acquiring information and mapping out daily participation characteristics, strengths, and challenges of children and adolescents with CD, while adhering to a GFD. The CD-Chart responds to aspects of everyday life with CD that have been unaddressed as yet by other available tools. Understanding these characteristics, in addition to following the medical and nutritional management guidelines of CD [4], can contribute to promote self-management of the GFD, health, well-being, and QOL by facilitating participation [54]. 

Additional studies are needed to further explore and validate the CD-Chart in children and adolescents with CD with bigger samples and in diverse cultures. Implementation of the CD-Chart in additional chronic food-related health conditions that requires self-management strategies may be beneficial and could be explored. The possibility to document each individual’s characteristics by means of the CD-Chart could lead to future development of intervention procedures and diverse implementation options.

## Figures and Tables

**Table 1:** Exploratory factor analysis of the CD-Chart.

Items	Factor 1: Social Environment	Factor 2: Close Family Environment
5. Participation in food served at a friend	0.756	
7. Participation in eating treats handed out by the teacher	0.705	
4. Participation in eating out with friends	0.658	
8. Participation in special food activities at school	0.587	
2. Participation in extended family meals/events	0.448	
3. Participation in meals during family vacations (local and abroad)		0.747
1. Preparing meals at home		0.703

**Table 2:** Comparison of means and standard deviations between CD group and control group for the four CD-Chart dimensions.

CD-Chart Dimensions	CD Group (*n* = 30)	Control Group (*n* = 30)			
	M (SD)	M (SD)	*F*(1,58)	*p*	η *p*^2^
Activity (scale = 0–1)	0.84 (0.12)	0.91 (0.09)	6.22	0.02	0.10
Frequency (scale = 1–4)	1.74 (0.29)	1.74 (0.24)	0.14	0.71	
Preference (scale = 1–10)	7.15 (1.41)	8.28 (0.91)	13.46	0.001	0.19

CD = celiac disease.

**Table 3:** Comparison between groups with and without CD across the four CD-Chart dimensions (MANOVA).

Factor/Component and Dimension	CD Group (*n* = 30)	Control Group (*n* = 30)	*F*(1,58)	*p*	η *p*^2^
	M (SD)	M (SD)			
Social environment					
Activity	0.91 (0.13)	0.99 (0.06)	8.12	0.008	0.12
Frequency	1.73 (0.39)	1.88 (0.25)	3.28	0.07	0.05
Preference	7.12 (1.68)	8.44 (1.16)	12.49	0.001	0.18
Close family environment					
Activity	0.97 (0.13)	1.00 (0.00)	2.07	0.155	
Frequency	2.05 (0.51)	1.95 (0.51)	0.57	0.455	
Preference	8.03 (1.73)	8.17 (1.23)	0.119	0.732	
Trip environment					
Activity	0.73 (0.25)	0.74 (0.25)	0.029	0.865	
Frequency	1.18 (0.61)	1.06 (0.22)	0.875	0.355	
Preference	6.18 (2.28)	7.90 (1.48)	9.64	0.003	0.18

CD = celiac disease.

**Table 4:** Comparison of means and standard deviations of six CD-Chart dimensions between children (8–11) and adolescents (12–18) with CD.

	Celiac Disease				
CD-Chart Dimensions	Children (*n* = 61)	Adolescents (*n* = 65)			
	M (SD)	M (SD)	*F*(1,124)	*p*	η *p*^2^
Activity (scale = 0–1)	0.81 (0.09)	0.92 (0.09)	45.60	<0.001	0.27
Frequency (scale = 1–4)	1.40 (0.31)	1.61 (0.25)	16.72	<0.001	0.12
Preference (scale = 1–10)	7.72 (1.02)	7.00 (1.45)	10.09	0.002	0.08
Preparation (scale = 0–1)	0.88 (0.06)	0.89 (0.05)	1.51	0.221	
Involvement (scale = 1–5)	3.01 (0.64)	3.52 (0.53)	24.06	<0.001	0.16
Self-determination (scale = 1–3)	1.90 (0.27)	2.11 (0.22)	22.64	<0.001	0.15

**Table 5:** Comparison between children (8–11) and adolescents (12–18) with CD across the six CD-Chart dimensions (MANOVA).

	Celiac Disease				
Factor/Component and Dimension	Children (*n* = 61)	Adolescents (*n* = 65)	*F*(1,124)	*p*	η *p*^2^
	M (SD)	M (SD)			
Social environment					
Activity	0.95 (0.09)	0.95 (0.10)	0.034	0.854	0.00
Frequency	1.69 (0.42)	1.73 (0.37)	0.449	0.504	0.01
Preference	7.64 (1.42)	7.06 (1.66)	4.34	0.039	0.03
Preparation	0.99 (0.04)	0.99 (0.48)	0.380	0.538	0.01
Involvement	3.14 (0.69)	3.64 (0.63)	17.87	<0.001	0.13
Self-determination	1.96 (0.28)	2.19 (0.28)	22.86	<0.001	0.16
Close family environment					
Activity	0.98 (0.09)	0.98 (0.06)	0.388	0.535	0.01
Frequency	1.78 (0.57)	2.11 (0.46)	12.73	0.001	0.09
Preference	8.26 (1.39)	7.89 (1.65)	1.76	0.187	0.01
Preparation	0.59 (0.19)	0.58 (0.20)	0.011	0.918	0.00
Involvement	2.45 (1.13)	2.97 (1.05)	7.04	0.009	0.05
Self-determination	1.74 (0.49)	1.99 (0.31)	12.12	0.001	0.09
Trip environment					
Activity	0.53 (0.12)	0.84 (0.25)	44.56	<0.001	0.33
Frequency	0.62 (0.31)	0.87 (0.29)	14.69	<0.001	0.14
Preference	6.85 (2.24)	5.75 (2.55)	4.29	0.041	0.05
Preparation	1.00 (0.00)	1.00 (0.00)	-	-	-
Involvement	2.79 (1.11)	3.60 (0.78)	16.94	<0.001	0.16
Self-determination	1.73 (0.52)	1.91 (0.42)	3.38	0.069	0.04
